# Genome-wide identification, characterization and expression pattern analysis of APYRASE family members in response to abiotic and biotic stresses in wheat

**DOI:** 10.7717/peerj.7622

**Published:** 2019-09-11

**Authors:** Wenbo Liu, Jun Ni, Faheem Afzal Shah, Kaiqin Ye, Hao Hu, Qiaojian Wang, Dongdong Wang, Yuanyuan Yao, Shengwei Huang, Jinyan Hou, Chenghong Liu, Lifang Wu

**Affiliations:** 1 Key Laboratory of High Magnetic Field and Ion Beam Physical Biology, Hefei Institutes of Physical Science, Chinese Academy of Sciences, Hefei, China; 2 University of Science and Technology of China, Hefei, China; 3 Anhui Province Key Laboratory of Medical Physics and Technology, Hefei Institutes of Physical Science, Chinese Academy of Sciences, Hefei, China; 4 Biotechnology Research Institute, Shanghai Academy of Agricultural Sciences, Shanghai, China

**Keywords:** Wheat, APYRASE, Abiotic and biotic stress, Expression pattern, Enzymatic activity

## Abstract

*APYRASE*s, which directly regulate intra- and extra-cellular ATP homeostasis, play a pivotal role in the regulation of various stress adaptations in mammals, bacteria and plants. In the present study, we identified and characterized wheat *APYRASE* family members at the genomic level in wheat. The results identified a total of nine APY homologs with conserved ACR domains. The sequence alignments, phylogenetic relations and conserved motifs of wheat APYs were bioinformatically analyzed. Although they share highly conserved secondary and tertiary structures, the wheat APYs could be mainly categorized into three groups, according to phylogenetic and structural analysis. Additionally, these APYs exhibited similar expression patterns in the root and shoot, among which *TaAPY3-1*, *TaAPY3-3* and *TaAPY3-4* had the highest expression levels. The time-course expression patterns of the eight *APY*s in response to biotic and abiotic stress in the wheat seedlings were also investigated. *TaAPY3-2*, *TaAPY3-3*, *TaAPY3-4* and* TaAPY6* exhibited strong sensitivity to all kinds of stresses in the leaves. Some *APY*s showed specific expression responses, such as *TaAPY6* to heavy metal stress, and *TaAPY7* to heat and salt stress. These results suggest that the stress-inducible *APY*s could have potential roles in the regulation of environmental stress adaptations. Moreover, the catalytic activity of TaAPY3-1 was further analyzed in the *in vitro* system. The results showed that TaAPY3-1 protein exhibited high catalytic activity in the degradation of ATP and ADP, but with low activity in degradation of TTP and GTP. It also has an extensive range of temperature adaptability, but preferred relatively acidic pH conditions. In this study, the genome-wide identification and characterization of *APY*s in wheat were suggested to be useful for further genetic modifications in the generation of high-stress-tolerant wheat cultivars.

## Introduction

Wheat is one of the most important crops grown around the world. However, constant pollution and overfertilization has exposed wheat cultivation to severe heavy metal and salt stresses. Fungal diseases such as Fusarium head blight, rust and powdery mildew also have threatened wheat yield improvement. Thus, the promotion of stress-resistant wheat could significantly improve yields. With recent fast developments in genome-editing and gene transformation technologies, it is becoming much easier to generate stress-resistant crop cultivators with these molecular tools. Nevertheless, investigating stress-regulatory networks, and identifying and characterizing of stress-related genes were our preliminary missions. As the genome information of many plant species has been clarified in recent years, the systematic genome-wide study of stress-related family genes using bioinformatic tools has become available. In wheat, transcription factors such as *MYB* (He et al., 2012; Zhang et al., 2012), *WRKY* (Ning et al., 2017; Niu et al., 2012), *No Apical Meristem* (*NAC*) (Xia et al., 2010) and *Dehydration Response Element-Binding* proteins (*DREB*) (Pellegrineschi et al., 2004) have been characterized as stress-related gene families. Meanwhile, many genes in wheat have shown specific responses under various stresses: for example, the *TRIHELIX* gene family under salt and cold stress (Xiao et al., 2019), Mitogen-activated protein kinase (*MAPK*) and *Catlase* (*CAT*) genes under osmotic stress (Dudziak et al., 2019), and *Glucose-6-Phosphate Dehydrogenase* (*G6PDH*) gene under salt stress (Nemoto & Sasakuma, 2000). Overexpression of stress-responsive genes from wheat could lead to a significant increase of stress tolerance, such as *TaASR1* in drought stress (Hu et al., 2013), *TaCIPK29* and *TaAQP8* in salt stress (Deng et al., 2013; Hu et al., 2012), *TaFER-5B* in heat and other stresses (Zang et al., 2017), *TaAQP7* in cold stress (Huang et al., 2014), and *TaWRKY44* in multiple abiotic stress (Wang et al., 2015). Thus, the identification of stress-responsive genes in wheat could help improve stress tolerance by using molecular strategies.

Environmental stresses can significantly elevate extra-cellular ATP levels, which further lead to the initiation of cell death and apoptosis (Cao et al., 2014). Cellular ATP level is tightly controlled by the GDA1-CD39 nucleoside phosphatase family, which widely exist in plants, animals, fungi and bacteria (Da’Dara et al., 2014; Matsumoto, Yamaya & Tanigawa, 1984; Tong et al., 1993). APYRASEs (APYs), a class of nucleoside triphosphate diphosphohydolases (NTPDases), play an important role in maintaining NTP homeostasis (Chiu et al., 2015). APYs can generally be divided into ecto- and endo-APY, according to their subcellular locations (Dunkley et al., 2004; Matsumoto, Yamaya & Tanigawa, 1984; Thomas et al., 1999; Tong et al., 1993). Ecto-APYs are localized on the cell surface while endo-apyrase are usually on Golgi, endoplasmic reticulum (ER) and intracellular vesicles (Leal et al., 2005). Some ecto-APYs have membrane-spanning domains at their N- and C-terminal, and usually have glycosylation on the amino acid, which is important for correct protein folding, membrane targeting, cellular allocation and enzyme activity (Knowles, 2011; Murphy & Kirley, 2003; Smith & Kirley, 1999; Wu, Choi & Guido, 2005). In contrast to ATPases that use Mg^2+^ as a co-factor, APYs can use a variety of divalent co-factors, including Ca^2+^, Mg^2+^, Mn^2+^ and Zn^2+^ (Yang, 2011). The cellular ATP level not only plays a pivotal role in supplying energy, but it also regulates various cellular processes related to abiotic stress responses, including Na^+^/H^+^ exchange, vacuolar Na+ distribution, K^+^ homeostasis, reactive oxygen (ROS) species regulation, and salt-responsive expression of K^+^/Na^+^ homeostasis and plasma membrane reparation (Sun et al., 2012a). Thus, cellular ATP homeostasis regulated by APYs is important for maintaining normal cell function.

So far, seven *APY* members have been identified and functionally characterized in *Arabidopsis* (Chiu et al., 2015). AtAPY1 and AtAPY2, which are located in the Golgi, are involved in the regulation of pollen germination, root growth and stomata closure (Chiu et al., 2015; Iris et al., 2003; Yang et al., 2015). Although AtAPY1 and AtAPY2 are endo-APYs, their mutation could cause significant elevation of extracellular ATP (eATP) (Hui et al., 2014; Wu et al., 2007), demonstrating that the intracellular-located APYs can also regulate eATP homeostasis. *AtAPY6* and *AtAPY7* also play pivotal roles in pollen development through the regulation of polysaccharide synthesis (Yang et al., 2013). Recently, *APY*s were found to be involved in the regulation of stress responses (Clark et al., 2014). Overexpression of *PeAPY2* in *Arabidopsis* leads to significant cleavage of reactive oxygen species (ROS) (Sun et al., 2012a), making them more tolerant to abiotic stresses. In some other species, *APY*s were also found to be directly involved in the regulation of biotic and abiotic stress resistance, such as drought and salt tolerance in *Populus euphratic* (Shurong et al., 2015; Sun et al., 2012b), pathogen resistance in pea and tobacco (Sharma, Morita & Abe, 2014; Tomonori, 2013), and water logging response in soybean (Alam et al., 2010), suggesting the pivotal role of *APY* in the regulation of stress adaptation. However, the molecular mechanism still largely remained unclear.

Due to the novel functions of *APY* in stress responses, the identification and functional characterization of *APY* family genes in crops could provide new targets for the improvement of stress tolerance via genetic modifications. Currently, there is still a lack of APY-related wheat studies. In this study, we identified the *APY* members in wheat at the genomic level using bioinformatic tools. We then performed a comprehensive characterization and phylogeny of the *TaAPY*s using bioinformatic and biochemical methods. The time-course expression pattern of these *APY* genes in response to various abiotic and biotic stresses was investigated. Additionally, *in vitro* enzymatic analysis was also performed. Conclusively, these results provide valuable insights in the bioinformatic and functional characteristics of the *APY* gene family in wheat, which would further benefit molecular breeding aimed at generating the stress-tolerant wheat cultivars.

## Material and Methods

### Screening of gene sequences

For identification of APY gene family members in the wheat genome, the amino acid sequence of seven *Arabidopsis thaliana* APYs directly obtained from TAIR (https://www.arabidopsis.org/) were used to blast against the wheat transcriptome and genome databases (Appels, Eversole & Feuillet, 2018) using the tBLASTN program with an e-value of 1 × e^−50^ as the threshold. The APY candidates were accepted only if the protein contained the conserved Apyrase Conserved Region (ACR) (Steinebrunner et al., 2000).

### Sequence alignment and phylogenetic analyses of APYs

The protein sequences of APYs from other species were obtained from the NCBI database. The sequence alignment and phylogenic analysis were both carried out using MEGA5 software, with maximum likelihood method with 1,000 bootstrap replicates and other parameters set as default, as shown previously (Jones, Taylor & Thornton, 1992; Tamura et al., 2011). The protein theoretical molecular weight (MW) and isoelectric point (IP) were predicted using online tools (http://au.expasy.org/tools). The exon/intron structure analysis was carried out by comparing the APY CDSs and their corresponding genomic sequences using the Gene Structure Display Server, as previously described (Hao & Qiao, 2018).

### Structure analysis of TaAPYs gene and amino acid sequences

The DNAman software was used to analyze the conservation property of CDS and amino acid (AA) sequences. The secondary structures of these proteins were predicted using the online tool NPS@SOPMA (https://npsa-prabi.ibcp.fr/) with default settings. The structure analysis included the percentage of each amino acid, the position and the alpha helix number, Beta Bridge, and Random coli, shown in different colors. The motif analysis was carried out using the MEME motif analysis with motif number set as 16 and other parameters as default. The membrane spanning motif method was analyzed using the online tool TMHMM (Jianyi, Ambrish & Yang, 2013) with default settings. The 3D models of tertiary structures were simulated using the Swiss-Model (https://www.swissmodel.expasy.org/) which is based on the automatic ExPASy (Expert Protein Analysis System) web server (Bertoni et al., 2017; Guex, Peitsch & Schwede, 2010; Pascal, Marco & Torsten, 2011; Waterhouse et al., 2018; Waterhouse et al., 2016).

### Abiotic and biotic stress treatment

Two-week-old wheat seedlings (Yangmai 158) were used for stress treatment. As previously described, 300 mM NaCl, 42 °C, 200 mM CdCl_2_, and 300 mM mannitol were separately used as salt, heat, heavy metal and drought treatment (Ni et al., 2018). The wheat leaf and root were collected at 1, 3, 6, 12 and 24 h post treatment. The *Bgt* spores were inoculated on the wheat leaves as previously described (Liang et al., 2019). The leaf sample was taken at 24, 48, 72 and 96 h post inoculation. The samples were immediately frozen in liquid nitrogen and stored at −80 °C. Each sample was prepared with three biological replicates.

### RNA isolation and quantitative real-time PCR

Total RNA was isolated using The Plant RNA kit (Omega, Shanghai, China). The RNA quantity and quality were determined using gel electrophoresis and a Scandrop spectrophotometer (Analytikjena, Jena, Germany), and cDNA was synthesized using the TransScript one-step gDNA Removal and cDNA synthesis SuperMix (Transgen, Beijing, China). qPCR was carried out using the SYBR Green PCR Kit (Qiagen, China) on the Lightcycler96 system (Roche, Swiss). The qPCR program was set as follows: preheating: 95 °C for 10 min, one cycle; Amplification: 95 °C for 10 s, 60 °C for 20 s, and 72 °C for 20 s, 45 cycles; Melting curve: 95 °C for 2 min, 60 °C for 30 s, then continuously increased to 95 °C as previously described (Ni et al., 2018). Raw data were calculated using the software given in the Lightcycler96 system. The primer information is listed in Table S3.

### Vector construction, recombinant protein expression and protein purification

The CDS of *TaAPY3-1*, with a removed region of the membrane spanning domain, was cloned intro the pET22a vector. The expression vector was then transformed into *Escherichia coli* BL21. The transformed cells were cultured in Luria-Bertani (LB) broth at 4 °C until the OD600 reached 0.8, and then were induced with 0.5 mM isopropyl beta-D-1-thiogalactopyranoside (IPTG). The induced cells were cultured at 37 °C for 5 h. The cells were then harvested by centrifugation at 10,000 ×g and resuspended with 20 ml lysis buffer (20 mM Tris, 500 mM NaCl, pH 7.6). The cells were then lysed by sonication as previously described (Ye et al., 2015).

The recombinant protein was further isolated from the denatured inclusion body using the Inclusion Body Protein Extraction Kit (Shengong, China). Briefly, the denatured protein was renatured by urea gradient dialysis in 500 ml renaturing buffers (10 mM Tris–HCl, 100 mM NaCl, 2 mM reduced glutathione, 0.2 mM oxidized glutathione, pH 8.5). The concentrations of urea in the renaturing buffers were 6.0, 4.0, 2.0, 1.0, and 0 M, respectively. Every dialysis step was carried out at 4 °C for 12 h. After the refolding process, the insoluble protein was removed by centrifugation at 10,000 ×g for 30 min at 4 °C. The amount and purity of recombinant protein were assessed using the BCA Protein Assay Kit (Shengong, China).

### Protein catalytic activity assay

The reaction system was set as: 50 mM Tris–HCl, 8 mM CaCl_2_, 0.25 ng/ul BSA, 2.5 mM DTT, 150 mM NaCl, and 0.05% Tween 20 (100 µl) as previously described (Dong et al., 2012). Then, 10 mM ATP (or ADP, TTP, CTP and GTP) and 4 µg purified recombinant APY were added into this system. The catalytic activity was analyzed under different conditions (temperature, pH, substrates, and ions) using the Phosphate Assay Kit (Jiancheng, China).

## Results

### Genome-wide identification and phylogenetic analysis of the APY family members in wheat

The protein sequences of the seven *Arabidopsis* APYs and the conserved ACR domains were used as the query sequences to BLAST against the recently published wheat genome and transcriptome database (Appels, Eversole & Feuillet, 2018). After careful validation of the candidates, a total of 27 APY members were identified with top hits for the AtAPY homologs (AtAPY1-7) in the wheat genome. These wheat APY candidates exhibited high sequence similarity and all contained five Apyrase Conserved Region (ACR) domains (Fig. S1). The 27 wheat *APYs* were further divided into nine groups, each group with three homologs located at different genome sets (A, B and D) (Table 1). The CDS information of these APYs is listed in Table S1. Based on the sequence similarity to the *Arabidopsis APY* homologs, the identified wheat *APY*s were separately named as *TaAPY1*, *TaAPY2*, *TaAPY3-1*, *TaAPY3-2*, *TaAPY3-3*, *TaAPY3-4*, *TaAPY5*, *TaAPY6* and *TaAPY7*. As shown in Table 1, the *APY* genes were predicted to encode 430 to 706 amino acids in length, with putative molecular weights ranging from 46.446 to 77.557 kDa, and the protein isoelectric points (PIs) from 5.93 to 9.2 (Table 1). As the three copies of the APYs from different wheat genome sets (A, B and D) had very high CDS sequence similarity (Table 1), the APYs from genome set A were used in the following bioinformatic and biochemical analysis.

**Table 1:** Characteristics of the APY members in wheat.

**Name**	**Gene ID**	**Protein length (AA)**	**CDS length (bp)**	**MW (kDa)**	**PI**	**Exon number**	**CDS similarity**
TaAPY1	TraesCS4A01G131300.1	485	1458	52.225	5.93	10	
	TraesCS4B01G173300.1	485	1458	52.261	6.05	10	98
	TraesCS4D01G175400.1	485	1458	52.25	6.34	9	98
TaAPY2	TraesCS2A01G102100.1	457	1374	48.91	6.68	9	
	TraesCS2B01G119200.1	459	1380	49.08	6.68	9	97
	TraesCS2D01G101500.1	469	1410	50.034	7.04	9	98
TaAPY3-1	TraesCS5A01G532000.1	462	1389	49.471	6.05	7	
	TraesCS4B01G363700.1	462	1389	49.555	6.36	7	95
	TraesCS4D01G357100.1	463	1392	49.493	6.22	7	95
TaAPY3-2	TraesCS5A01G547700.1	457	1374	48.963	8.89	6	
	TraesCS4B01G381600.1	430	1293	46.446	8.81	6	94
	TraesCS4D01G357100.1	452	1359	49.036	6.06	10	83
TaAPY3-3	TraesCS7A01G160900.1	454	1365	49.196	5.96	7	
	TraesCS2B01G025000.1	449	1350	49.178	6.76	7	95
	TraesCS2D01G020200.2	448	1347	49.979	7.07	7	95
TaAPY3-4	TraesCS7B01G004400.2	435	1308	47.008	9.29	9	
	TraesCS7D01G100000.1	431	1296	46.720	9.29	9	95
	TraesCSU01G095000.1	437	1314	47.258	9.40	9	94
TaAPY5	TraesCS6A01G105900.1	502	1509	54.652	8.81	8	
	TraesCS7B01G178800.1	465	1398	51.287	8.96	8	96
	TraesCS7D01G280900.1	447	1344	49.209	8.02	6	96
TaAPY6	TraesCS6A01G105900.2	340	1023	36.015	8.13	8	
	TraesCS6B01G135200.1	502	1509	54.54	9.05	8	92
	TraesCS6D01G094400.2	502	1509	54.535	8.86	8	94
TaAPY7	TraesCS1A01G288900.1	706	2121	77.499	9.2	2	
	TraesCS1B01G298200.1	706	2121	77.557	9.19	2	98
	TraesCS1D01G287900.1	706	2121	77.472	9.2	2	98

**Notes.**

CDS coding sequence AA amino acid MW molecular weights PI protein isoelectric points

**Figure 1:**
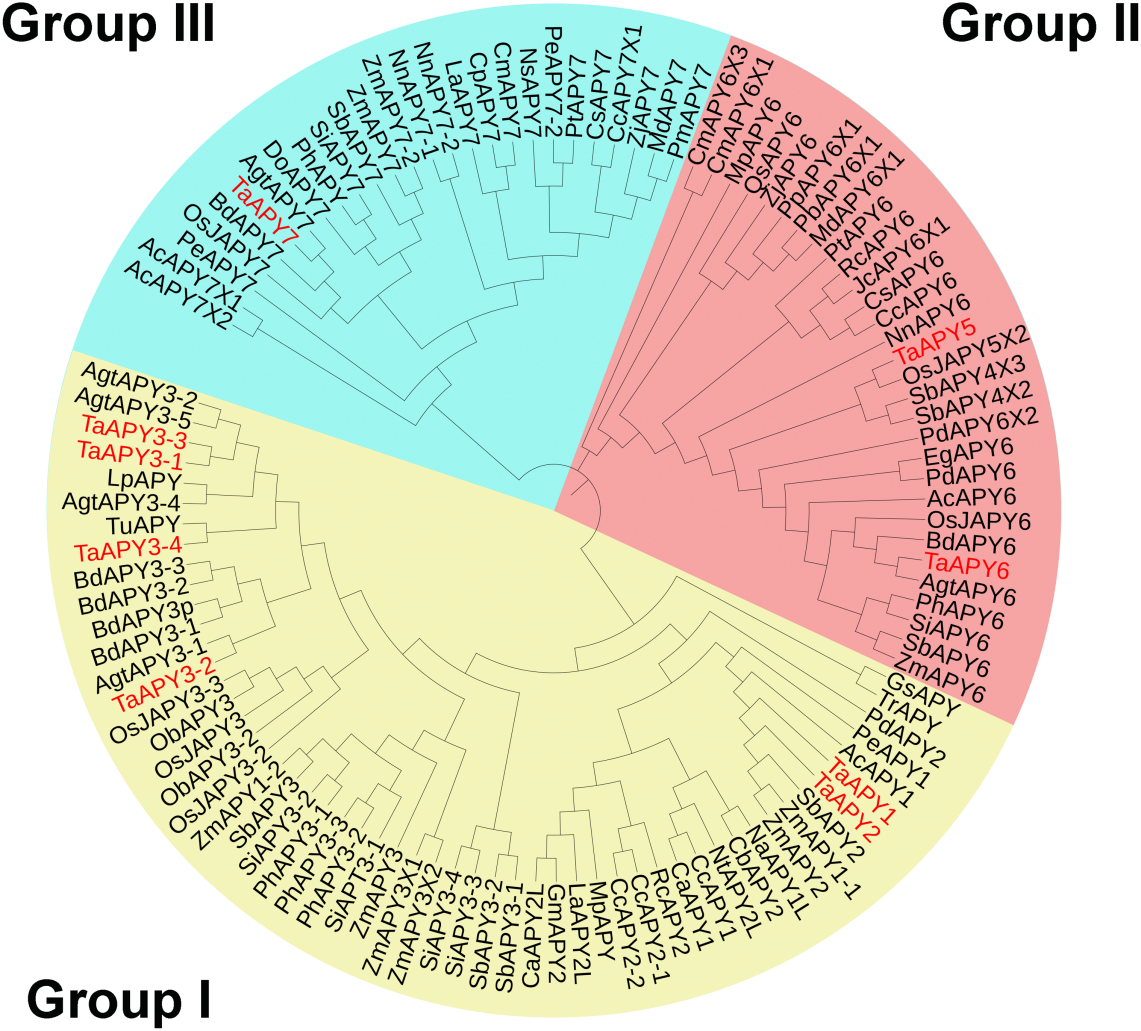
Phylogenetic analysis of the putative APYs in wheat and other plant species. The phylogenetic tree was created using the MEGA5 software with maximum Likelihood method. Bootstrap values for 1,000 replicates were indicated. Genes were separated into three groups and marked with different colors (Group I–III), according to the categorization in the phylogenetic tree.

To investigate the evolutionary relationships among the APYs, a phylogenetic tree was constructed using the APY homologs from *Arabidopsis thaliana*, *Zea mays*, *Oryza sativa*, *Aegilops tauschii*, *Phoenix dacylifera*, and some other species (Table S2). The APYs can be mainly divided into three distinct groups: I, II, and II. Specifically, TaAPY1, TaAPY2, TaAPY3-1, TaAPY3-2, TaAPY3-4, and TaAPY3-3 were clustered in Group I, TaAPY5 and TaAPY6 in Group II, and TaAPY7 in Group III (Fig. 1).

### Gene structure and conserved motif analysis of the APY genes in wheat

**Figure 2:**
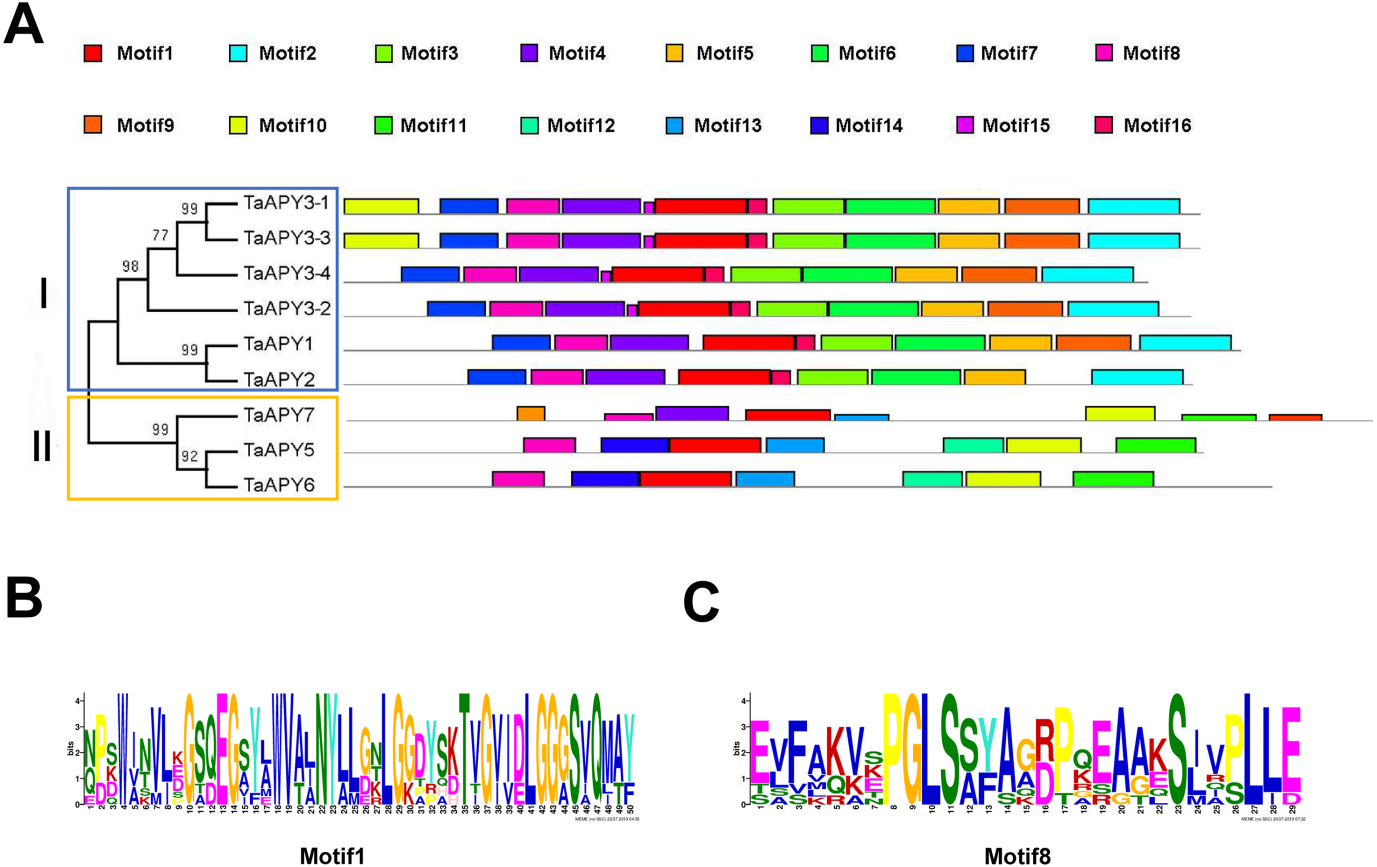
Conserved motif analysis of the wheat APYs. (A) The motif analysis of the eight APYs was carried out by using the online software MEME suite 5.0.2. (B) and (C) The details of the conserved motifs (1 and 8) of the nine APYs were represented. Different motifs were represented in different colors in the protein.

**Figure 3:**
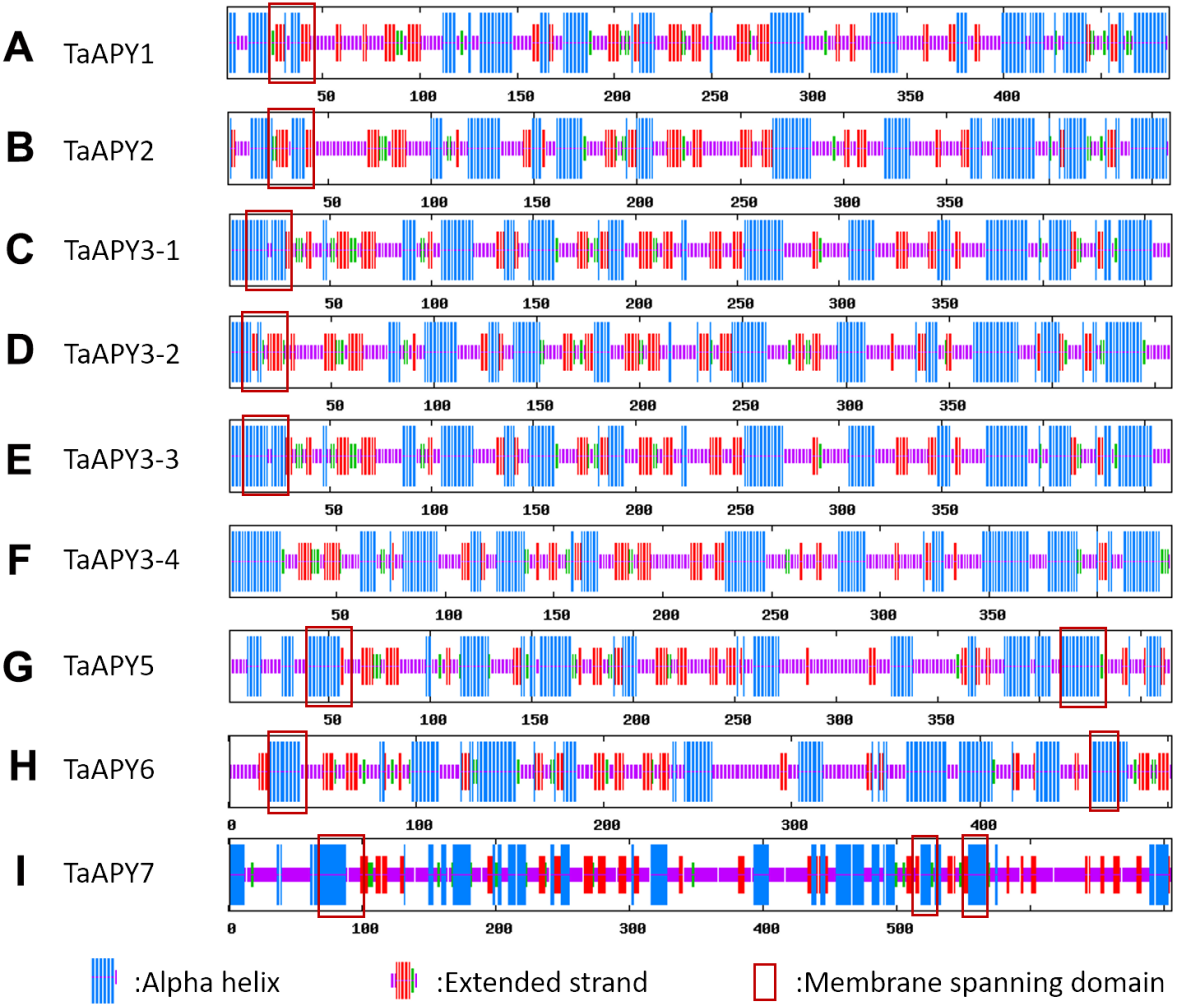
Secondary structure analysis of the nine wheat APYs (A–I). Alpha helix was colored in red, Extend strand in blue and random coli in purple. The cross membrane domain was predicted and marked with a red box.

**Figure 4:**
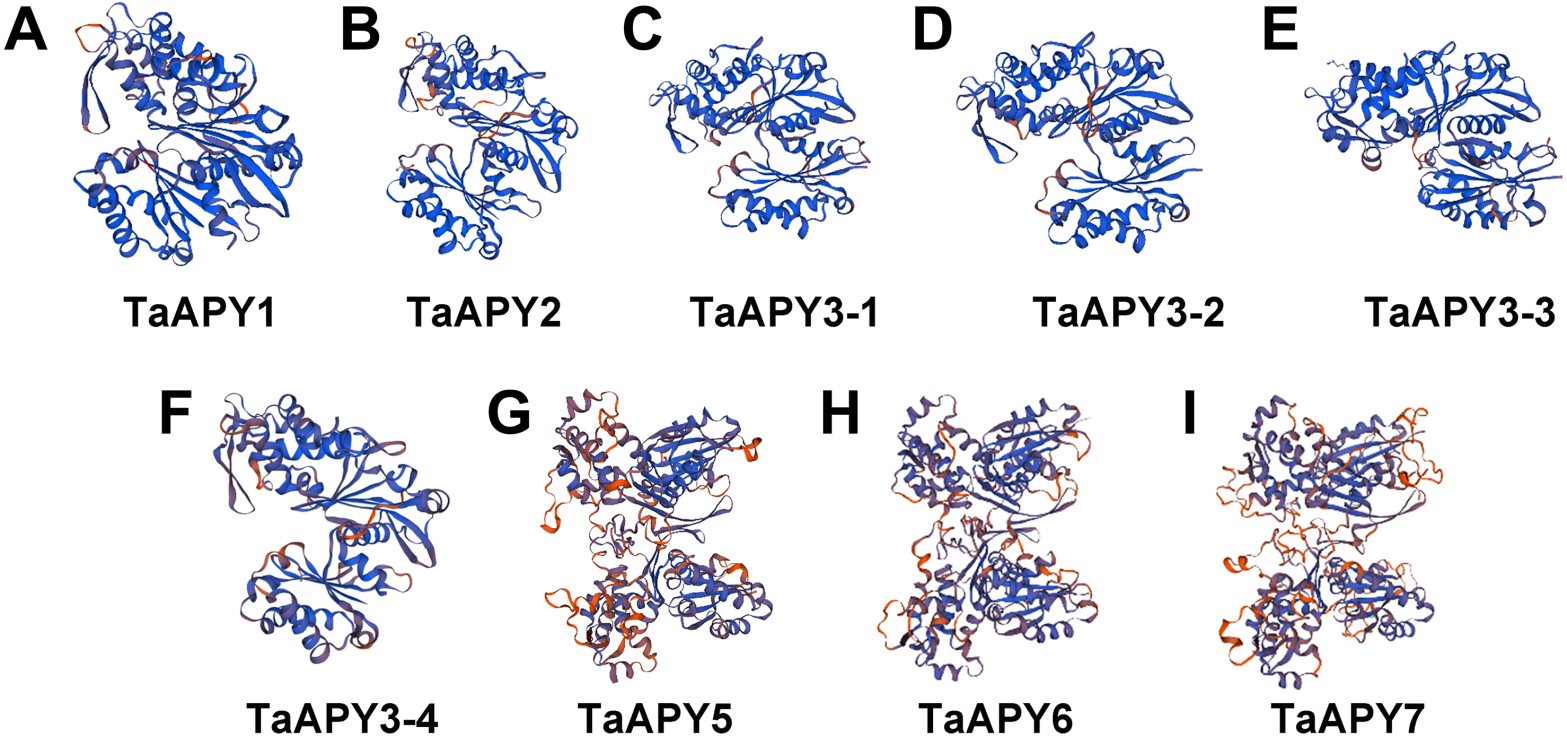
3D structure analysis of the nine wheat APYs. The structure models of the nine wheat APYs (A–I) were constructed using the Swiss-model website (https://www.swissmodel.expasy.org/).

To investigate the structural diversity of the APYs, the online structural analysis tool NPS @ SOPMA (Deléage, 2017) was used for conserved motif analysis. As showed in Fig. S1, the amino acid sequences of those nine APYs were highly conserved. Additionally, a total of 16 motifs can be detected among the APYs (Fig. S2). Generally, the APYs can be mainly divided into two groups (I, II) by the motif analysis. Nine wheat APYs all contained conserved motif 1 and 8 (Fig. 2). APYs in the same group had specific motifs, such as motif 2, 3, 16, 19 to group I, motif 12 and 13 to group II, motif 15 to TaAPY3, motif 10 to TaAPY3-1 and TaAPY3-3, and motif 5 to TaAPY7 (Fig. 2). Further, the membrane spanning motif (MSM) analysis showed that Group I APYs (TaAPY1, 2, 3-1, 3-2 and 3-3) were predicted to contain only one MSM at N-terminal, whereas Group II APYs (TaAPY5 and 6) had two MSMs, separately located at the N- and C-terminals (Fig. 3). Interestingly, it was predicted that TaAPY3-4 contained no MSMs, while TaAPY7 contained three MSMs (Fig. 3). To compare, the MSM of the seven APY members from *Arabidopsis* were also analyzed. The results showed that except for AtAPY5 and 7, which separately had two and three MSMs, the others only exhibited one MSM at the N-terminal, which was similar to the wheat APYs (Fig. S3). The membrane-spanning was closely related to protein allocation and transportation. Thus, these results provided important information of their potential cellular roles. Moreover, the 3D structure analysis of the nine proteins showed that TaAPY5, 6 and 7 contains four subunits while other TaAPYs only have two. Two similar subunits of the APY were linked with an extended strand surrounded by an alpha helix (Fig. 4), which was a signature character of the GDA1-CD39 nucleoside phosphatase super family. Based on the results of 3D protein structure simulation (Fig. 4), the nine wheat APYs can also be divided into two groups: Group I (TaAPY1, 2, 3-1, 3-2, 3-3 and 3-4), and Group II (TaAPY5, 6 and 7), similar to the motif analysis. Although TaAPY5, TaAPY6, and TaAPY7 were categorized into different groups in the phylogenetic trees (Fig. 1), they shared high 3D structure similarity (Fig. 4).

### Gene expression pattern of the APYs in wheat seedlings in response to abiotic and abiotic stresses

**Figure 5:**
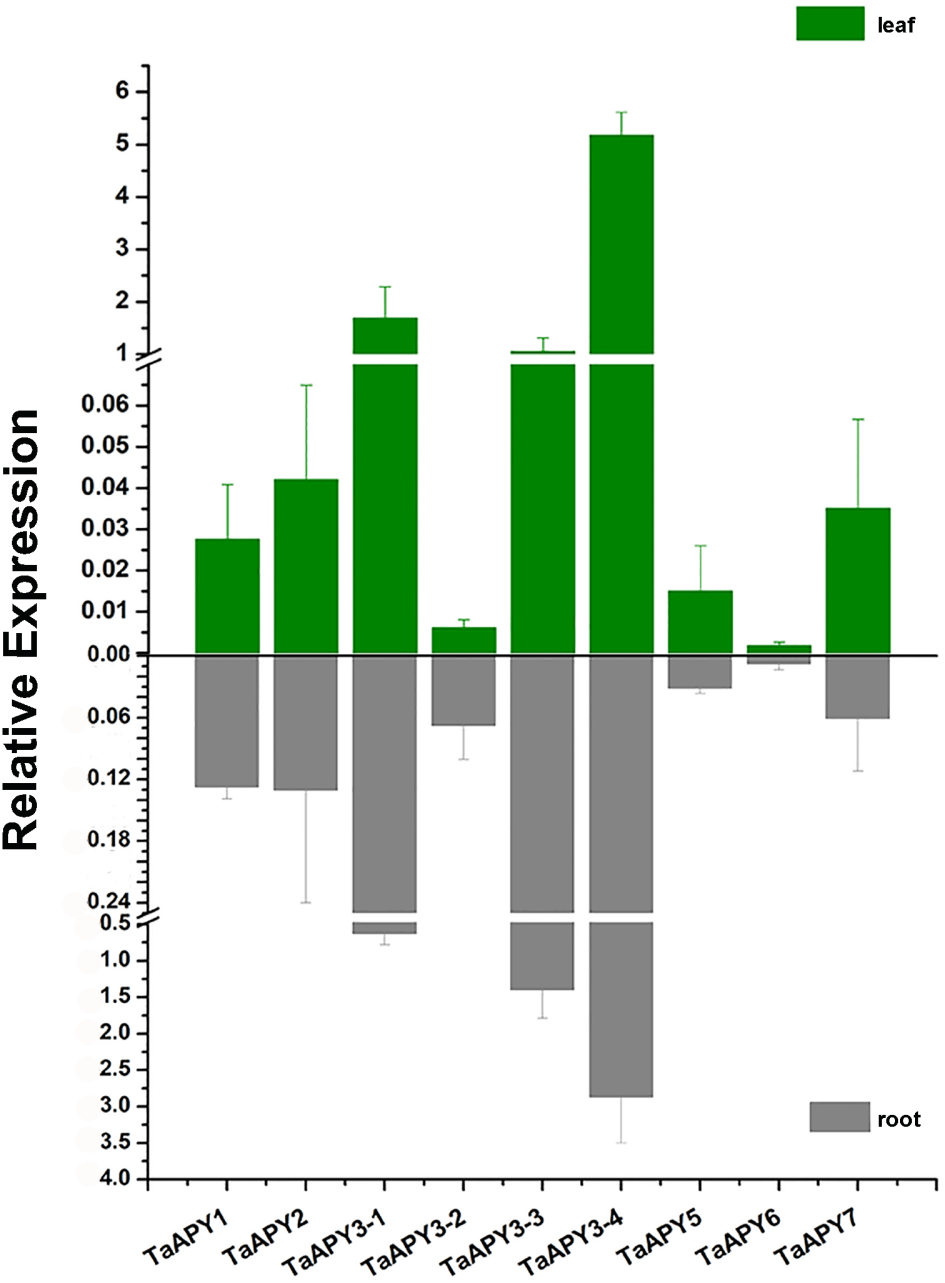
Expression pattern of the nine *TaAPY*s in the root and leaf of the 10-d-old wheat seedlings. *TaACT* was used as internal control. Data are presented as means ± SD of three biological replicates.

To investigate the expression pattern of the nine *APY*s in wheat, the gene expression in shoot and root, and their responses to abiotic stresses (salt, heat, heavy metal and osmotic stresses) were further analyzed using quantitative real-time PCR. The results showed that all nine *APY*s had similar expression levels at the seedling leaf and root, with *TaAPY3-4*, *TaAPY3-1* and *TaAPY3-3* having the highest expression level, and *TaAPY6* the lowest both in the shoot and root (Fig. 5), suggesting *TaAPY3-1*, *TaAPY3-3* and *TaAPY3-4* could be the predominant *APY*s in wheat.

**Figure 6:**
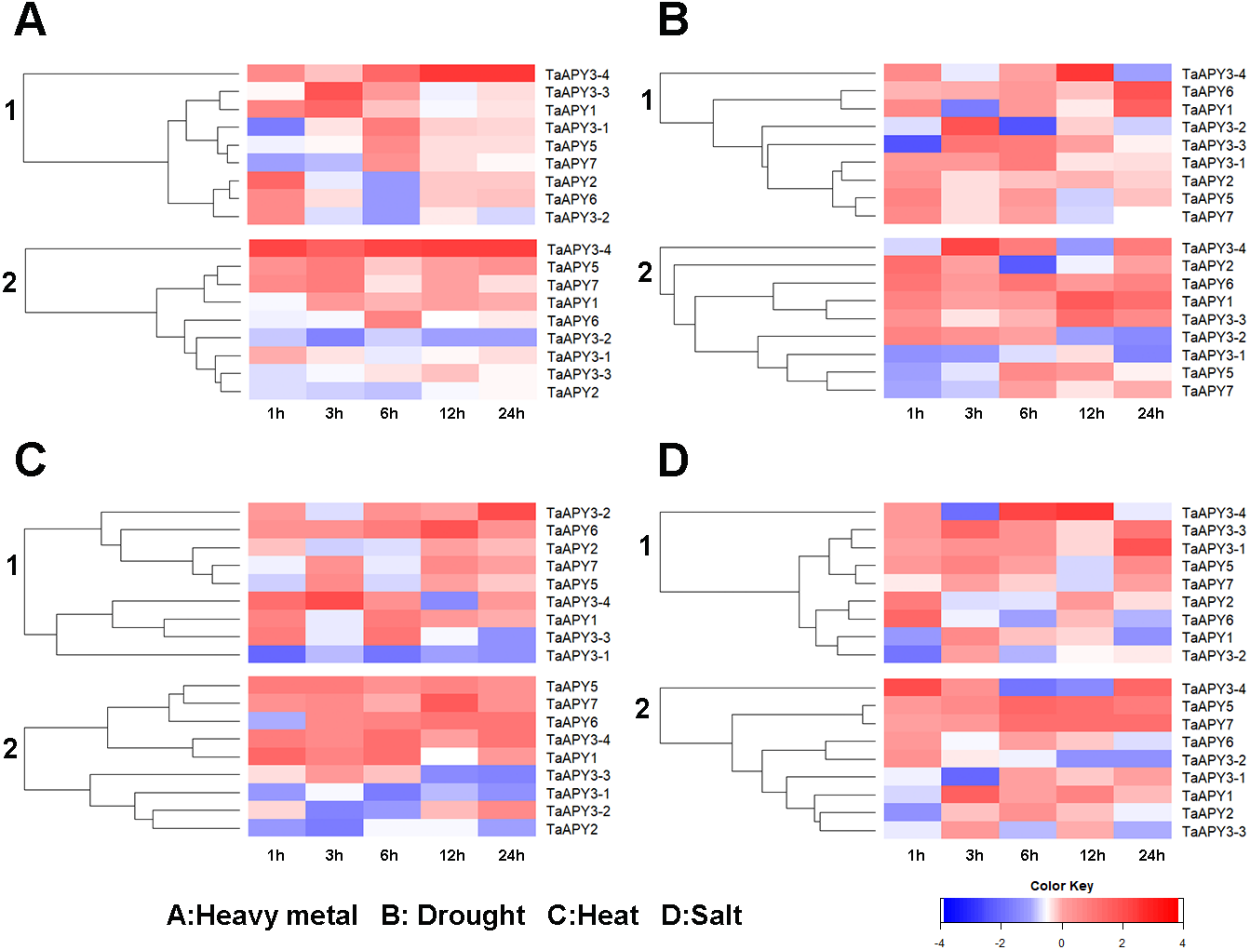
Expression pattern of the wheat *APY*s in response to the abiotic stresses. (A) Heavy metal (200 mM CdCl_2_). (B) Drought (300 mM mannitol). (C) Heat (42 °C). (D) Salt (300 mM NaCl). 1, leaf. 2, root. *TaACT* was used as internal control. Different colors represented decreased or decreased expression level.

Previous studies have shown that several *APY*s could be involved in the regulation of abiotic stress adaptation (Clark et al., 2014). Thus, we further investigated the time-course expression profiles of *TaAPY*s in the leaf and root of wheat seedlings in response to different abiotic stresses. The results showed that in leaves, most of the *TaAPY*s could be upregulated after being subjected to cadmium treatment (Fig. 6A). Specifically, *TaAPY3-4* exhibited high expression level both in the leaf and root under cadmium stress, while *TaAPY3-1* reached a peak at 6 h in the leaves. The expression of the other *TaAPY*s in the root was not as sensitive as in the leaf, and only *TaAPY6* exhibited a significant upregulation at 6 h (Fig. 6A). Further, mannitol treatment was used to produce an artificial drought stress condition in the wheat seedlings. The results showed that all the *TaAPY*s could be up-regulated within 24 h, among which, *TaAPY1*, *TaAPY3-4*, and *TaAPY6* reached an extremely high expression level in the leaves (Fig. 6B). On the contrary, very few genes exhibited significant changes at different times post-mannitol-treatment in the root (Fig. 6B), suggesting that these *TaAPY*s also regulated the drought responses in the shoot, not only in the root. Under heat stress, the expression of *TaAPY3-2*, *TaAPY3-4*, *TaAPY5*, *TaAPY6*, and *TaAPY7* began to increase both in the leaves and root at 12 h, with the highest increase fold of *TaAPY3-2* (Fig. 6C). For salt stress, the expression of all the *TaAPY*s significantly increased in the leaves at 12 h, but only *TaAPY1*, *TaAPY3-4*, *TaAPY7*, and *TaAPY5* were shortly up-regulated in the root, while others remained unaffected throughout (Fig. 6D).

**Figure 7:**
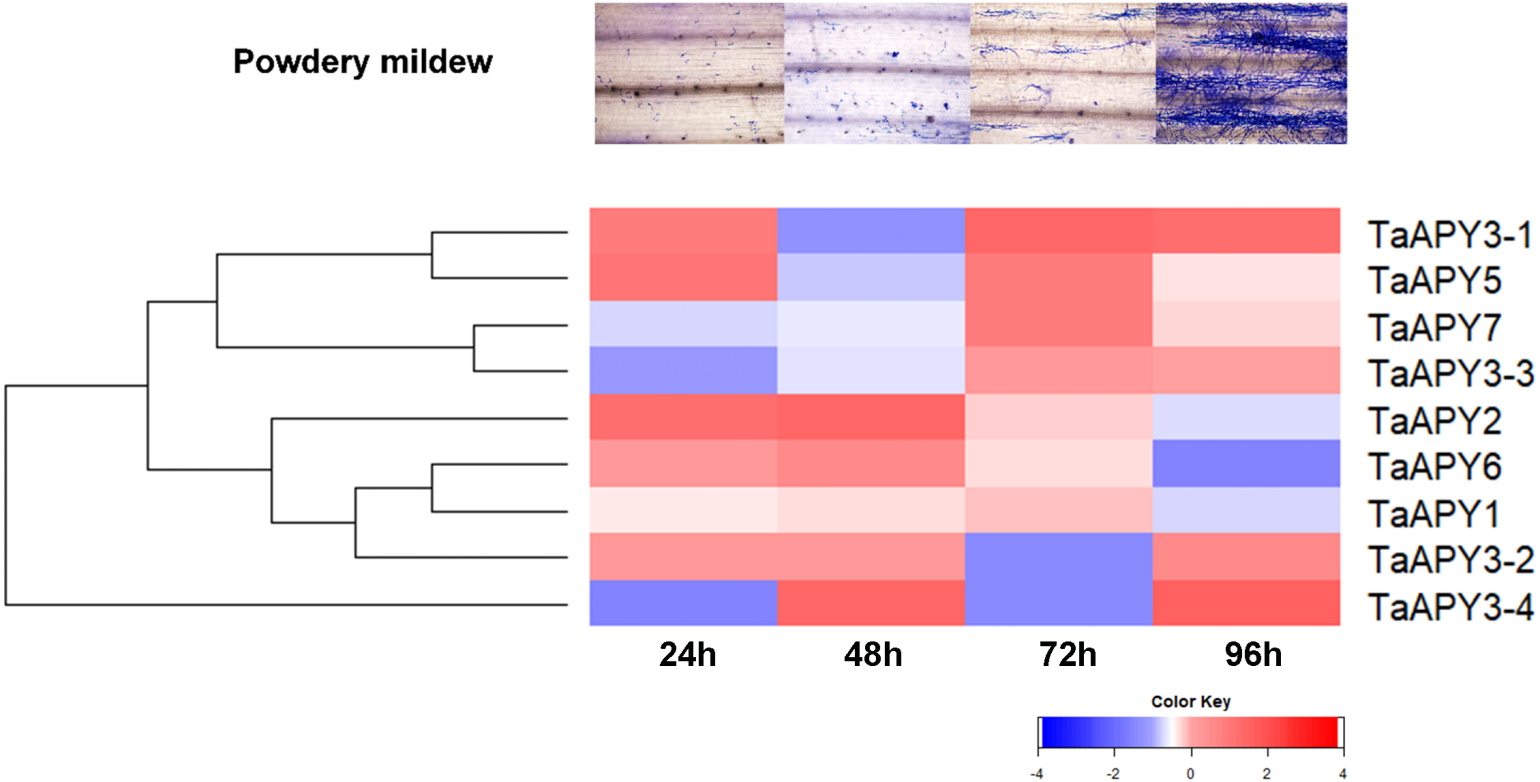
Expression pattern of the *APY*s in response to the *Bgt* infection. The expression of the *APY*s was analyzed separately at 24, 48, 72 and 96 h post *Bgt* infection. *TaACT* was used as the internal control. Green and red colors represented decreased or decreased expression level.

To test for biotic stress, powdery mildew pathogen was used in the wheat seedlings. The results showed that seven *TaAPY* s (except *TaAPY1*) showed significant sensitivity to *Blumeria graminis* infection. Specifically, the significant up-regulation of *TaAPY2*, *TaAPY3-1*, *TaAPY3-2*, *TaAPY5* and *TaAPY6* could be detected at the pre-penetration stage (24 h), whereas *TaAPY3-3*, *TaAPY3-4*, and *TaAPY7* were significantly up-regulated at the late infection stages (Fig. 7). These results suggested that the wheat APYs could be involved in the regulation of biotic stress responses. However, different APYs may have diverse roles at different infection stages.

### Enzymatic analysis of the recombinant APY3-1 in wheat

**Figure 8:**
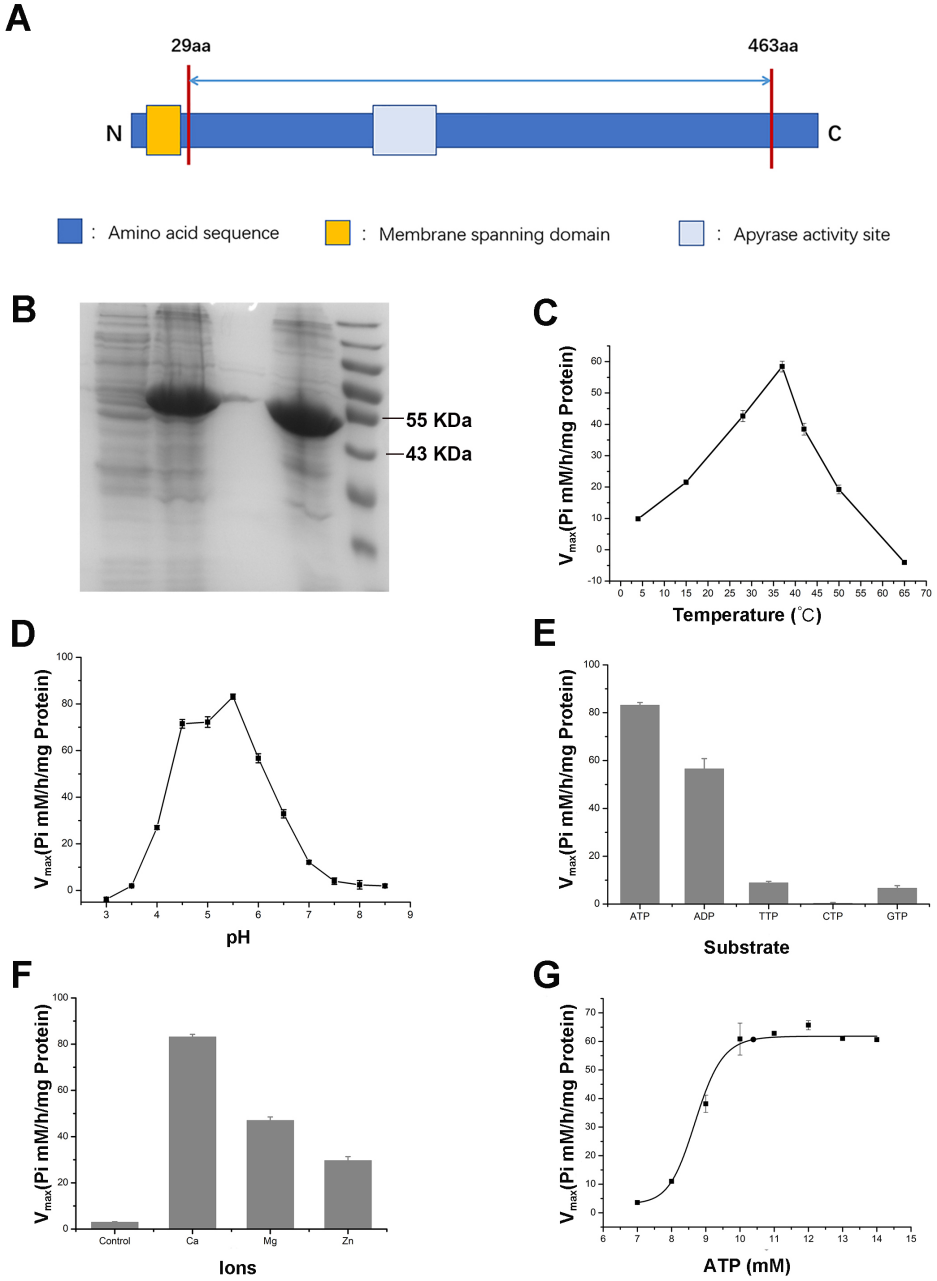
Enzymatic activity analysis of recombinant TaAPY3-1. (A) Scheme of the purified TaAPY3-1 without the membrane spanning domain. (B) SDS-PAGE analysis of the protein purification. (C) Enzymatic activity of TaAPY3-1 in degradation of ATP under different temperature. (D) Activity of TaAPY3-1 under different pH. (E) Enzymatic activity of TaAPY3-1 in degradation of ATP, ADP, TTP, CTP and GTP. (F) Effects of different ions (Ca^2 +^, Mg^2 +^ and Zn^2 +^) on the enzymatic activity of TaAPY3-1 in degradation of ATP. (G) Enzymatic activity analysis of TaAPY3-1 with different concentrations of ATP. Data are presented as means ± SD of three biological replicates.

To further validate the enzymatic activity of the wheat APYs, the recombinant APY3-1, without the cross-membrane domain, was cloned and purified using the *Escherichia coli* expression system (Figs. 8A and 8B). The production of the inorganic phosphate in the system was used to determine ATP hydrolyzation as previously described (Dong et al., 2012). The results showed that the recombinant TaAPY3-1 exhibited high enzymatic activity with a relatively wide temperature range from 25 to 52 °C, and had the highest catalytic activity at 37 °C (Fig. 8C). Further, the APY3-1 had relatively high enzyme activities at the acid conditions, with maximal activity detected at pH 4.5 to 5.5 (Fig. 8D). Moreover, the hydrolyzation efficiency of APY3-1 on different substrates was also evaluated. The results showed that the APY3-1 exhibited slightly lower enzymatic activity when degrading the ADP compared with ATP, but had very low activity during the degradation of TTP, GTP and CTP, suggesting that TaAPY3-1 has a high substrate preference (Fig. 8E). Moreover, it has been demonstrated that all NTPDase family members require divergent metal ions as cofactors for their enzymatic activity. Thus, we also evaluated the enzymatic activity of different ions in degrading the ATP. The results showed that Ca^2+^ was the most effective cofactor. The preference order was as follows: Ca^2+^ > Mg^2+^ > Zn^2+^ (Fig. 8F). Without the ions, the APY3-1 had no enzymatic activity, suggesting that apyrase activity is dependent on ions as cofactors, with a preference for Ca^2+^. The catalytic activity of TaAPY3-1 can reach a peak of Vmax = 61 (Pi µM/h/µg protein), Km = 8.7 mM under the most appropriate conditions (37 °C, pH 5.5, 8 mM Ca^2+^) (Fig. 8G). Conclusively, these results suggested that the APY3-1 had high and specific ATP and ADP degradation activities, which was consistent with the functions of the APY homologs reported in other species.

## Discussion

APYRASEs (APYs) play a key role in maintaining regular cell growth and stress responses (Clark et al., 2014; Kiwamu et al., 2010; Clark & Roux, 2018). Stresses can cause significant eATP efflux from the cell, leading to increased ROS accumulation and further induced cell apoptosis (Jeter et al., 2004; Kiwamu et al., 2010; Song et al., 2006; Vadim et al., 2010). Thus, the efficient cleavage of the overdosed eATP by APYs could be important for the prevention of stress-induced cell apoptosis. Recent research has shown that overexpression of *APY* could significantly inhibit ROS production and could promote stress resistance (Shurong et al., 2015), proving that *APY*s could be very useful targets for the improvement of stress tolerance. With accurate sequencing and assembly of the bread wheat genome (Appels, Eversole & Feuillet, 2018), the identification of *APY* family members in wheat became available, which was useful for studying their gene functions. In this paper, we identified and characterized the *TaAPY* family members at the genomic level. The results showed that a total of nine *APY* genes, all containing conserved ACR domains, were identified in the wheat genome (Table 1). The identification and characterization of the *APY*s could provide valuable insights in understanding the physiological and biochemical functions of wheat APYs in stress responses.

A total of seven *APY* members have been identified in *Arabidopsis* (Tsan-Yu et al., 2015). In this study, nine APY homologs were identified in the wheat genome (Fig. 1), and were further divided into three groups based on their phylogenetic relationship and three-dimensional structures (Fig. 4). It was postulated that the transmembrane character could be associated with the subcellular locations of the proteins (Chiu et al., 2012; Knowles, 2011; Zimmermann, 2010). In mammals, all four ecto-APYs contained both N- and C-terminal transmembrane domains, while others were endo-APYs with only C-terminal transmembrane domains (Knowles, 2011; Tsan-Yu et al., 2015). TaAPY5 and TaAPY6 contained both N- and C-membrane spanning motifs (MSMs), and TaAPY7 had three MSMs (Fig. 3). In *Arabidopsis*, although AtAPY6 and AtAPY7 contained both N- and C-terminal MSM (Fig. S3), they were endo-APYs which were localized to the ER, similar to other AtAPYs (Tsan-Yu et al., 2015). These results suggested that MSM characters could not be considered as the only markers for protein subcellular locations. Although *APY1* and *APY2* in *Arabidopsis* proved to be inter-cellularly located, their mutation can cause significant elevation of the eATP level (Hui et al., 2014; Wu et al., 2007), suggesting that endo-APYs can also regulate eATP homeostasis in plants.

The investigation of the gene expression pattern in response to stresses could help in identifying gene function (Wang et al., 2018; Wang et al., 2019). In wheat, overexpression of the stress-responsive genes, such as *TaMYB73* (He et al., 2012), *TaASR1* (Hu et al., 2013), *TaCIPK29* and *TaAQP8* (Deng et al., 2013; Hu et al., 2012), *TaAQP7* (Huang et al., 2014), *TaFER-5B* (Zang et al., 2017), and *TaWRKY44* (Wang et al., 2015), could significantly improve stress tolerance. Thus, the significantly increased *APY* expression in response to stresses indicated that some *APY*s could be directly or indirectly involved in the regulation of stress adaptation in wheat (Figs. 6 and 7). In *Arabidopsis*, it has been reported that the expression of *AtAPY5*, *6* and *7*, but not of *AtAPY3* and *4*, was very sensitive to stresses, such as wounding and drought (Yang, 2011). In *Medicago truncatula*, all four *MtAPY1* s can be significantly up-regulated in response to various stresses in root (Cohn et al., 2001; Navarro-Gochicoa et al., 2003). In wheat, the nine *APY*s exhibited various expression patterns in response to different stresses, and even varied in different organs (Figs. 5, 6 and 7). Generally, the expression of most *APY*s (including *TaAPY1*, *TaAPY2*, *TaAPY3-1*, *TaAPY3-3*, *TaAPY3-4*, *TaAPY6* and *TaAPY7*) was up-regulated in the leaf under drought stress, and barely up-regulated in root except in *TaAPY5* and *TaAPY6* (Fig. 6). For salt stress, genes exhibited significant up-regulation in the root, except in *TaAPY3-1*, *TaAPY3-2*, and *TaAPY3-3* (Fig. 6). Among all these *APY*s, *TaAPY3-4* exhibited strong responses to the above mentioned stresses. Specifically, we also found that the expression of *TaAPY2*, *TaAPY3-1*, and *TaAPY5* exhibited significant up-regulation at 24 h after powdery mildew inoculation (Fig. 7), suggesting their potential role in the regulation of primary defense in response to powdery mildew. Conclusively, wheat APYs were mostly stress-responsive, and some exhibited stress specificity.

In this study, the recombinant protein of TaAPY3-1was purified and its enzymatic activity was further evaluated under different conditions. Unlike the ecto-APY (NTPDase1) from human lymphocytes (Leal et al., 2005), the TaAPY3-1 protein exhibited high stability within a much wider temperature range from 4 °C to 60 °C, and reached the highest activity at 37 °C (Fig. 8). The adaptation to the wide range of temperatures indicated its potential in the regulation of thermo- and cold-stress responses. Further, relative alkaline conditions were required for the enzymatic activities of NTPDase1 (Leal et al., 2005) and APY from *Sergentomyia schwetzi*, whose activity is maintained over a pH range of 6.5 to 9.0 (Volfova & Volf, 2018), while the appropriate reaction pH for TaAPY3-1 was only 4.5 to 6. The reaction buffer pH was over 7, and almost diminished the enzymatic activity of TaAPY3-1 (Fig. 8). The pH preferences of different proteins could be due to their cellular locations, whereas the pH of different cell compartments is different. In *Arabidopsis*, AtAPY3 exhibited relatively high enzymatic activity in hydrolyzing ATP, UTP, GTP and CTP, and it can also hydrolyze ADP and GDP (Tsan-Yu et al., 2015). Although TaAPY3-1 and AtAPY3 were homologous proteins, TaAPY3-1 only exhibited high enzymatic activity to ATP and ADP, low activity to TTP and GTP, and no activity to CTP. Similar to other reports in different species, APYs could have different preferences for substrates. For instance, two APYs, GDA1 and YND1 in yeast, have a preference for GDP. LALP1 in human has a preference for UTP, GTP and CTP (Shi et al., 2001) and the APY in sand fly can digest both ATP and ADP (Volfova & Volf, 2018). These results demonstrate that homologs in different species might function differently, and TaAPY3-1 in wheat is an ATP/ADP-specific APY. It has been proved that the activity of TaAPYs relies on different metal ions such as Ca^2+^, Mg^2+^ and Zn^2+^. Different apyrases have different preferences for these divalent cations (Guranowski et al., 1991). However, ATPases only use Mg^2+^ as a co-factor (Komoszynski & Wojtczak, 1996), and our results showed that TaAPY3 has the highest preference for Ca^2+^, but could also use Mg^2+^ and Zn^2+^ as co-factors. Since the function of apyrase in stress responses is Ca^2+^mediated (Sun et al., 2012b), and Ca^2+^ is a second messenger for stress responses, the interactions between apyrase and calcium signaling might be pivotal for stress adaptation.

## Conclusion

In this study, we identified and characterized the APY family members in wheat at the genome-wide level. Phylogenetic, structural and expression analyses provided a theoretical basis for further functional study and for genetic improvement during molecular breeding for a generation of stress-resistant wheat cultivars.

##  Supplemental Information

10.7717/peerj.7622/supp-1 Figure S1 Amino acid sequence alignment and conserved motif analysis of the wheat APYs

10.7717/peerj.7622/supp-2 Figure S2 Information of the conserved motifs detected among the nine wheat APYs

10.7717/peerj.7622/supp-3 Figure S3 Membrane spanning motif (MSM) analysis of the *Arabidopsis* APYs

10.7717/peerj.7622/supp-4 Data S1 Raw data

10.7717/peerj.7622/supp-5 Table S1 CDS sequence of the 27 *APY* candidates

10.7717/peerj.7622/supp-6 Table S2 List of the species used in the phylogenetic analysis

10.7717/peerj.7622/supp-7 Table S3 Information of the primers used in this study

## Additional Information and Declarations

### Data Availability

The following information was supplied regarding data availability: The raw data is available in the Supplemental Files.
