# Supplementary figures and images for: Genome-wide identification, characterization and expression pattern analysis of APYRASE family members in response to abiotic and biotic stresses in wheat
Source: PeerJ. 2019 Sep 11;7:e7622. doi: 10.7717/peerj.7622 (PMC6744936; doi:10.7717/peerj.7622)

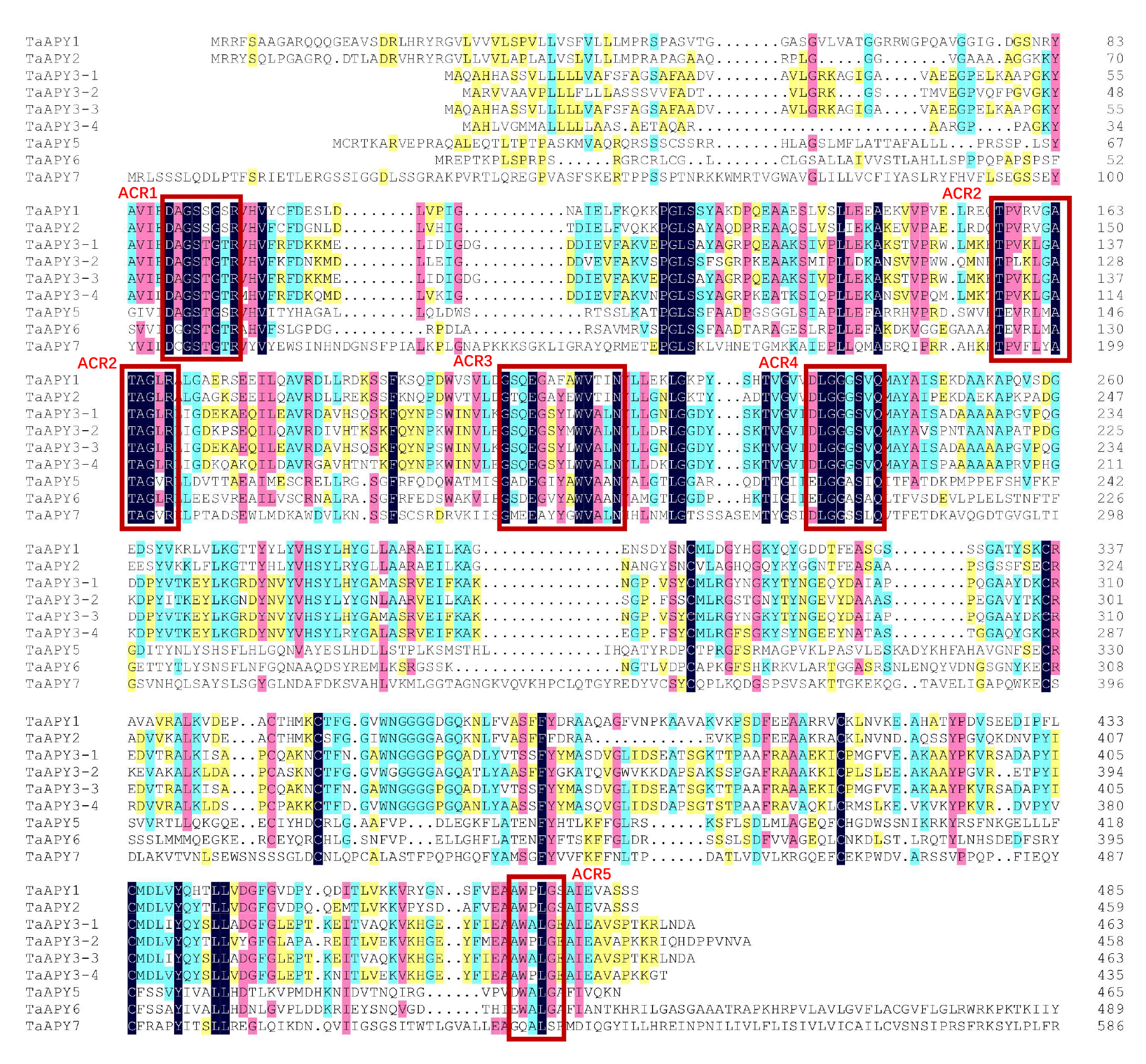

Supplement: Figure S1 [file peerj-07-7622-s001.png]

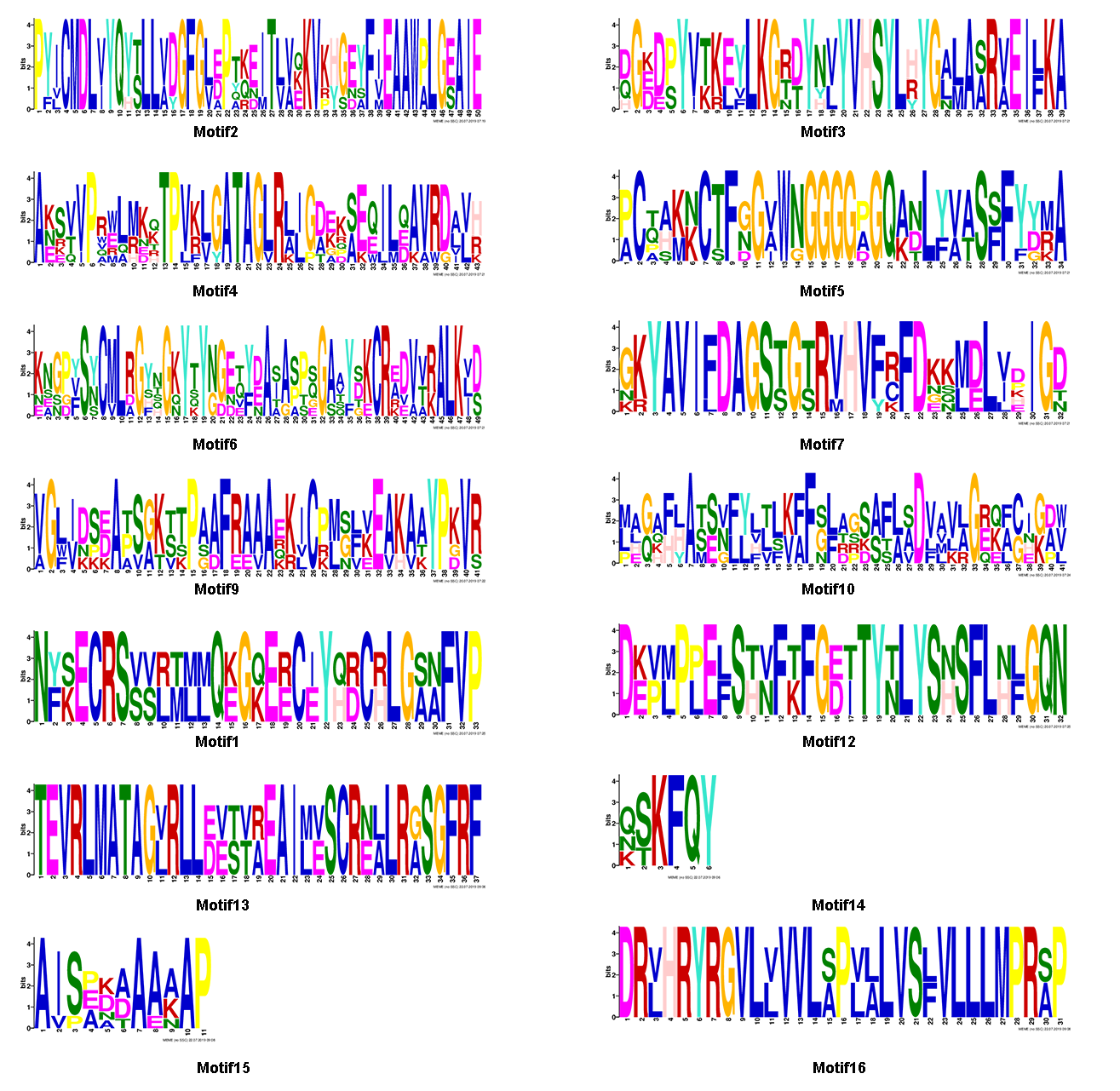

Supplement: Figure S2 [file peerj-07-7622-s002.png]

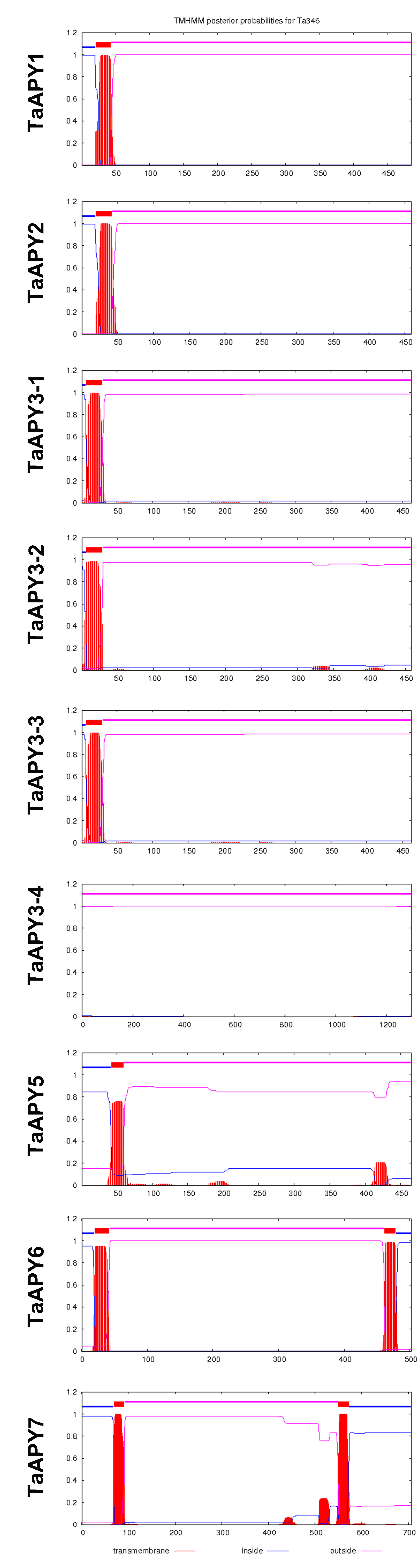

Supplement: Figure S3 [file peerj-07-7622-s003.png]
