# Supplementary material for: Genome-wide identification, characterization and expression pattern analysis of APYRASE family members in response to abiotic and biotic stresses in wheat
Source: PeerJ. 2019 Sep 11;7:e7622. doi: 10.7717/peerj.7622 (PMC6744936; doi:10.7717/peerj.7622)
Supplement: Data S1 [file peerj-07-7622-s004.doc]

**Raw data of experiments of manuscript**

**Figure 6**:

| Raw data for heavy metal treatment | | | | | | |
| --- | --- | --- | --- | --- | --- | --- |
| Tissue | Gene name | Time after treatment (h) | | | | |
|  |  | 1 | 3 | 6 | 12 | 24 |
| leaf | TaAPY3-1 | -1.67288 | -1.61598 | 2.817406 | 1.463728 | 0.851042 |
|  | TaAPY3-3 | -0.63666 | 1.311142 | 1.072582 | -0.12625 | 0.63745 |
|  | TaAPY3-4 | 0.259737 | -1.32838 | 3.910999 | 14.07991 | 10.07695 |
|  | TaAPY3-2 | 0.215596 | -2.03737 | -2.59725 | 0.687174 | -0.71582 |
|  | TaAPY1 | 0.459291 | 0.697695 | 0.046105 | -0.07762 | 0.578232 |
|  | TaAPY2 | 0.938062 | -1.95251 | -2.63274 | 1.475496 | 1.094567 |
|  | TaAPY7 | -1.19076 | -2.2779 | 1.375696 | 1.038063 | 0.194366 |
|  | TaAPY6 | 0.316573 | -1.51837 | -2.73688 | 1.526764 | 1.204 |
|  | TaAPY5 | -0.73731 | -1.75659 | 1.830848 | 1.105287 | 0.670014 |
| root | TaAPY3-1 | -1.98117 | -1.3848 | -1.25525 | -1.44517 | -1.36044 |
|  | TaAPY3-3 | -4.29111 | -1.75195 | -0.43884 | -0.25402 | -1.74551 |
|  | TaAPY3-4 | 5.980612 | 2.492369 | 7.810571 | 9.64643 | 7.43517 |
|  | TaAPY3-2 | -4.69733 | -3.70052 | -1.75052 | -3.75955 | -3.59186 |
|  | TaAPY1 | -3.71194 | -0.18922 | 0.380558 | 0.739165 | -0.37863 |
|  | TaAPY2 | -4.27111 | -2.26648 | -1.98231 | -1.70839 | -1.76367 |
|  | TaAPY7 | -0.20557 | 1.203488 | -0.4956 | 0.48972 | -1.35651 |
|  | TaAPY6 | -3.83369 | -1.80776 | 3.10834 | -1.53617 | -1.53845 |
|  | TaAPY5 | -0.35236 | 0.969653 | 0.081089 | 0.450337 | 1.095184 |

| Raw data for drought treatment | | | | | | |
| --- | --- | --- | --- | --- | --- | --- |
| Tissue | Gene name | Time after treatment (h) | | | | |
|  |  | 1 | 3 | 6 | 12 | 24 |
| leaf | TaAPY3-1 | -0.86431 | -1.38108 | 0.884209 | -0.2187 | 1.558401 |
|  | TaAPY3-3 | -4.93865 | -0.8806 | 0.881283 | 1.066649 | 1.23285 |
|  | TaAPY3-4 | -0.46907 | -2.03838 | -0.36191 | 7.985124 | -0.54313 |
|  | TaAPY3-2 | -2.38228 | -0.29028 | -4.11438 | 0.192539 | 0.233543 |
|  | TaAPY1 | -0.60112 | -2.76717 | -0.02532 | -0.25825 | 6.437539 |
|  | TaAPY2 | -0.79685 | -1.79346 | -0.58801 | 0.562553 | 1.689978 |
|  | TaAPY7 | -0.46189 | -1.8077 | -0.3578 | -1.26414 | 1.009063 |
|  | TaAPY6 | -1.44277 | -1.59761 | -0.06502 | 0.32964 | 7.281631 |
|  | TaAPY5 | -0.26213 | -1.80217 | 0.040306 | -1.4409 | 1.884787 |
| root | TaAPY3-1 | -3.58552 | -2.17834 | -3.79197 | -2.67835 | -1.7527 |
|  | TaAPY3-3 | -1.63528 | -0.40949 | -2.32725 | 1.010574 | -0.30907 |
|  | TaAPY3-4 | -2.95134 | 4.962839 | -0.20386 | -5.04086 | -0.01203 |
|  | TaAPY3-2 | -1.22189 | 1.037973 | -2.11141 | -4.81773 | -1.6567 |
|  | TaAPY1 | -1.15194 | 0.481281 | -1.51872 | 2.56483 | 0.125137 |
|  | TaAPY2 | -0.60717 | 0.34311 | -7.43283 | -3.46599 | -0.64127 |
|  | TaAPY7 | -3.25652 | -1.31469 | -1.83921 | -2.78281 | -0.68908 |
|  | TaAPY6 | -0.85801 | 0.898516 | 0.264467 | -1.34013 | -0.201 |
|  | TaAPY5 | -3.34233 | -1.00106 | -0.70493 | -1.1316 | -0.94367 |
| Raw data for heat treatment | | | | | | |
| Tissue | Gene name | Time after treatment (h) | | | | |
|  |  | 1 | 3 | 6 | 12 | 24 |
| leaf | TaAPY3-1 | -4.69603 | -2.81201 | -3.30844 | -1.51402 | -2.58052 |
|  | TaAPY3-3 | 0.036837 | -2.38948 | 2.285167 | -0.33844 | -2.63953 |
|  | TaAPY3-4 | 0.542892 | 1.779053 | 0.869355 | -2.36323 | 1.397079 |
|  | TaAPY3-2 | -1.088 | -2.48668 | 0.817897 | 0.931525 | 6.264039 |
|  | TaAPY1 | -0.07947 | -2.35838 | 1.994461 | 1.470413 | 0.629111 |
|  | TaAPY2 | -1.61225 | -2.62977 | -1.18492 | 1.147516 | 0.461624 |
|  | TaAPY7 | -2.33649 | -0.71172 | -1.00556 | 2.041647 | 0.902711 |
|  | TaAPY6 | -0.59432 | -0.88413 | 2.039955 | 5.055355 | 1.855506 |
|  | TaAPY5 | -2.65581 | -0.54664 | -1.24012 | 0.923957 | 0.213861 |
| root | TaAPY3-1 | -2.84855 | -1.92586 | -5.9463 | -0.94116 | -3.7352 |
|  | TaAPY3-3 | -1.97974 | -0.4378 | -1.88881 | -2.0655 | -4.20365 |
|  | TaAPY3-4 | -0.60526 | 0.136241 | 1.581488 | 0.970072 | 0.534839 |
|  | TaAPY3-2 | -1.92132 | -3.76495 | -4.68569 | 0.739632 | -0.24743 |
|  | TaAPY1 | -0.03873 | 0.548503 | 1.594201 | -0.06968 | -1.09422 |
|  | TaAPY2 | -2.86482 | -4.09913 | -2.93078 | -0.19508 | -3.31521 |
|  | TaAPY7 | -1.23452 | 0.324214 | -1.61108 | 4.537775 | -0.57978 |
|  | TaAPY6 | -2.702 | 0.057245 | 0.452707 | 3.162064 | 0.458035 |
|  | TaAPY5 | -0.57787 | 0.936487 | -0.60866 | 2.491313 | -0.66049 |

| Raw data for salt treatment | | | | | | |
| --- | --- | --- | --- | --- | --- | --- |
| Tissue | Gene name | Time after treatment (h) | | | | |
|  |  | 1 | 3 | 6 | 12 | 24 |
| leaf | TaAPY3-1 | -0.72455 | -0.3443 | 1.399169 | 1.138647 | 1.882975 |
|  | TaAPY3-3 | -0.61178 | 0.737104 | 1.470745 | 1.170289 | 1.376015 |
|  | TaAPY3-4 | -0.63409 | -2.62384 | 5.133239 | 8.983468 | 0.383979 |
|  | TaAPY3-2 | -2.14561 | -0.66906 | -1.08083 | 0.554067 | 0.524505 |
|  | TaAPY1 | -1.69182 | -0.11914 | 0.481554 | 1.152673 | -0.07577 |
|  | TaAPY2 | -0.13383 | -1.35044 | -0.51762 | 2.35122 | 0.55604 |
|  | TaAPY7 | -1.09498 | -0.61637 | 0.317748 | -0.15585 | 0.829955 |
|  | TaAPY6 | 0.236836 | -1.25047 | -1.4346 | 1.54011 | 0.182308 |
|  | TaAPY5 | -0.64805 | 0.084463 | 0.803103 | -0.18583 | 1.093882 |
| root | TaAPY3-1 | -4.76804 | -4.10308 | -0.73115 | -1.00544 | -1.01245 |
|  | TaAPY3-3 | -4.84265 | -1.34762 | -2.09308 | -0.76103 | -3.35251 |
|  | TaAPY3-4 | 2.731342 | -1.21323 | -3.43132 | -3.15765 | 1.573821 |
|  | TaAPY3-2 | -1.95816 | -2.11376 | -1.62953 | -2.8417 | -4.16814 |
|  | TaAPY1 | -5.14417 | 0.175868 | -0.76102 | 0.454941 | -1.50926 |
|  | TaAPY2 | -6.74797 | -1.82507 | -0.2078 | -0.93924 | -2.54128 |
|  | TaAPY7 | -2.76834 | -1.33545 | 0.978094 | 1.465923 | 1.083506 |
|  | TaAPY6 | -2.48085 | -2.2818 | -0.71962 | -0.96014 | -2.79636 |
|  | TaAPY5 | -2.42792 | -1.07234 | 1.154078 | 1.464418 | 0.61176 |

**Figure7**:

| Raw data for mildew powdery treatment | | | | | |
| --- | --- | --- | --- | --- | --- |
| Tissue | Gene name | Time after treatment (h) | | | |
|  |  | 24 | 48 | 72 | 96 |
| leaf | TaAPY3-1 | 3.08264 | -2.2258 | 2.124901 | 2.680525 |
|  | TaAPY5 | 3.330493 | -1.60419 | 1.319605 | 1.327904 |
|  | TaAPY7 | 0 | -1.36717 | 1.318069 | 1.385069 |
|  | TaAPY3-4 | -1.46694 | 1.242054 | -2.02031 | 2.938149 |
|  | TaAPY3-3 | -0.79365 | -1.4138 | 0.492586 | 1.641208 |
|  | TaAPY2 | 3.676237 | 1.200478 | -0.28687 | 1.032462 |
|  | TaAPY6 | 1.792798 | 0.08449 | -0.39284 | 0.331102 |
|  | TaAPY1 | 0.639269 | -0.96279 | -0.19125 | 0.987355 |
|  | TaAPY3-2 | 1.983374 | -0.38934 | -2.16631 | 2.05189 |

**Figure 8**:

panel (C) was generated with the following data(n=3):

| Temprature（℃） | V_max_（Pi mM/h/mg Protein） | SD for V_max_ |
| --- | --- | --- |
| 4 | 4.66428 | 0.4218 |
| 15 | 7.61787 | 0.3048 |
| 28 | 21.35761 | 0.54642 |
| 37 | 34.38099 | 1.7634 |
| 42 | 22.02426 | 0.20252 |
| 50 | 15.41201 | 1.13127 |
| 65 | 2.68467 | 0.15798 |

panel (D) was generated with the following data(n=3):

| pH | V_max_（Pi mM/h/mg Protein） | SD for V_max_ |
| --- | --- | --- |
| 3 | -3.73491 | 0.76698 |
| 3.5 | 1.94865 | 0.33048 |
| 4 | 27.00405 | 0.85423 |
| 4.5 | 71.47906 | 1.84253 |
| 5 | 72.22413 | 2.25689 |
| 5.5 | 83.13274 | 1.13103 |
| 6 | 56.72092 | 1.94445 |
| 6.5 | 32.8691 | 1.74823 |
| 7 | 12.0883 | 0.7616 |
| 7.5 | 4.02147 | 1.40024 |
| 8 | 2.42148 | 1.82915 |
| 8.5 | 1.96298 | 0.77466 |

panel(E) was generated with the following data(n=3):

| Substrate(NTP) | V_max_（Pi mM/h/mg Protein） | SD for V_max_ |
| --- | --- | --- |
| ATP | 83.13274 | 1.13103 |
| ADP | 56.54866 | 4.28148 |
| TTP | 8.89043 | 0.59134 |
| CTP | 0.31939 | 0.36028 |
| GTP | 6.62234 | 1.05674 |

panel(F) was generated with the following data(n=3):

| Ions | V_max_（Pi mM/h/mg Protein） | SD for V_max_ |
| --- | --- | --- |
| NONE | 2.9945 | 0.22624 |
| Ca^2+^ | 83.13274 | 1.13103 |
| Mg^2+^ | 47.02349 | 1.50559 |
| Zn^2+^ | 29.65904 | 1.66878 |

panel(G) was generated with the following data(n=3):

| ATP concentration(mM) | V_max_（Pi mM/h/mg Protein） | SD for V_max_ |
| --- | --- | --- |
| 7 | 3.713943 | 0.225899 |
| 8 | 10.93536 | 0.200187 |
| 9 | 40.16095 | 4.07703 |
| 10 | 64.94994 | 8.167066 |
| 11 | 62.80971 | 0.324813 |
| 12 | 65.72295 | 1.130606 |
| 13 | 61.28132 | 0.557873 |
| 14 | 60.98673 | 0.707493 |
